# Identification, classification, and transcription profiles of the B-type response regulator family in pear

**DOI:** 10.1371/journal.pone.0171523

**Published:** 2017-02-16

**Authors:** Junbei Ni, Songling Bai, Ling Gao, Minjie Qian, Linbing Zhong, Yuanwen Teng

**Affiliations:** 1 Department of Horticulture, Zhejiang University, Hangzhou, Zhejiang, PR China; 2 The Key Laboratory of Horticultural Plant Growth, Development and Quality Improvement, the Ministry of Agriculture of China, Hangzhou, Zhejiang, PR China; 3 Zhejiang Provincial Key Laboratory of Integrative Biology of Horticultural Plants, Hangzhou, Zhejiang, PR China; 4 Tonglu Extension Center of Agricultural and Forestal Technology, Tonglu, Zhejiang, PR China; Key Laboratory of Horticultural Plant Biology (MOE), CHINA

## Abstract

Type-B response regulators (B-RRs) are transcription factors that function in the final step of two-component signaling systems. In model plants, B-RRs have been shown to play important roles in cytokinin signal transduction. However, the functions of B-RRs in pear have not been well studied. In this report, we conducted a genome-wide analysis and identified 11 putative genes encoding B-PpRR proteins based on the published genome sequence of *Pyrus bretschneideri*. A phylogenetic tree of the *B-PpRR* family was constructed, and the motif distribution, chromosome localization, and gene structure of *B-PpRR* family genes were determined. Gene transcript profiles, which were determined from transcriptome data, indicated that *B-PpRR* genes potentially function during pear fruit development, bud dormancy, and light/hormone-induced anthocyanin accumulation. Treatment of the fruitlets of ‘Cuiguan’ pear (*Pyrus pyrifolia*), which never accumulates anthocyanin, with the cytokinin N-(2-chloro-4-pyridyl)- N′-phenylurea (CPPU) clearly induced anthocyanin accumulation. Anthocyanins accumulated in the skin of fruitlets by 16 days after CPPU treatment, along with the significant activation of most anthocyanin biosynthetic genes. Analyses of *B-PpRR* transcript levels suggested that *B-PpRR* genes mediated this accumulation of anthocyanins. These findings enrich our understanding of the function of *B-PpRR* genes in the physiological processes of pear.

## Introduction

Cytokinins are *N*^*6*^-substituted adenine derivatives that play essential roles in regulating plant growth and development. They have been shown to control cell division, vascular development, chloroplast biogenesis, stress tolerance, senescence, and differentiation of the shoot meristem, leaf, and root [1]. Cytokinin signal transduction is mediated by a two-component system based on a phosphotransfer cascade, which refers to phosphorylation on a nitrogen atom of a histidine (His) residue and on an acyl group of an aspartate (Asp) residue [2]. The two-component system was originally found in bacteria [3]. In *Arabidopsis*, there are three cytokinin receptors known as *Arabidopsis* histidine kinases (AHK), AHK2, AHK3, and AHK4/CRE1/WOL. The autophosphorylation of a conserved His residue in the kinase domain occurs after binding to cytokinins. The phosphate group is then transferred to a conserved Asp residue in the receiver domain of the receptors [1], and further to *Arabidopsis* histidine-phosphotransfer proteins (AHP). Finally, the phosphate group is transferred to the *Arabidopsis* response regulators (ARR) and releases their activities.

The ARR is the essential player in cytokinin signal transduction. Two types of ARRs (A-type and B-type) have been identified based on structure and function. Both the A-type and B-type RRs have a receiver domain at the N-terminal, but they differ at the C-terminal. The C-terminal of the B-type RRs (B-RR) has a long extension with a MYB-like DNA binding domain [4]. In total, 10 *A-ARRs* and 12 *B-ARRs* have been identified in the *Arabidopsis* genome. B-ARRs function as transcription factors that directly bind to the promoter of target genes and activate their transcription [2]. *A-ARRs* are direct targets of *B-ARRs* and negatively regulate the cytokinin signaling pathway [2]. Specifically, *A-ARRs* inhibit the activity of *B-ARRs*, forming a negative feedback loop in the cytokinin signaling pathway [1]. Overexpression of *ARR1*, one of the most studied *B-ARRs*, resulted in enhanced sensitivity to cytokinin [5], while the triple mutant *arr1*,*10*,*12* showed almost complete insensitivity to cytokinin. Single mutants were not significantly different from wild type, indicating that there is functional overlap among *B-ARRs* [6].

Studies on model plants such as *Arabidopsis* have revealed the important roles of B-RRs in regulating multiple biological processes, including cell division, differentiation in the root meristem, organ size [7–9], and anthocyanin accumulation [10]. Cytokinins have been shown to regulate a wide array of downstream responses through *ARR1/10/12*, indicating that they play the most important roles in cytokinin signal transduction. The triple mutant *arr1*,*10*,*12* with decreased cell division showed significant lower expression of a D-type cyclin, CycD3;1, which is implicated in the regulation of cell division by cytokinins [6, 11]. The inflorescence stems of the triple mutant *arr1 arr10 arr12* were narrower than those of wild type, and their siliques were much shorter [6]. Together, these findings demonstrated that *B-ARRs* regulate cell division and organ size via the cytokinin signaling pathway. It was also reported that the triple mutant *arr1 arr10 arr12* accumulated less anthocyanin than did wild type when treated with cytokinin. Thus, cytokinins may regulate fruit development and anthocyanin biosynthesis at least partly through B-type *RRs*.

Cytokinins are known to regulate fruit size in pear [12]. However, less is known about their functions in dormancy and coloration. To unravel the functions of cytokinins in pear development, in this study, B-type *RR* genes were isolated based on the published genome sequence of *Pyrus bretschneideri*. Phylogenetic and structural analyses classified the pear *B-RRs* into four main groups. Then, transcript profile analyses using RNA-seq data indicated that members of Group II play important roles in pear fruit development, bud dormancy, and light-induced anthocyanin accumulation. Experimental analyses revealed that *B-RRs* were involved in N-(2-chloro-4-pyridyl)- N′-phenylurea (CPPU)-mediated fruitlet coloration of *P*. *pyrifolia* cv. Cuiguan, providing further evidence that cytokinin stimulates anthocyanin accumulation. These findings enrich our understanding of the potential functions of *B-RR* genes during pear fruit development, and provide basic information for further studies on the molecular mechanism of cytokinins in anthocyanin accumulation and floral bud dormancy.

## Materials and methods

### Fruit bagging treatment

Fruit of red Chinese sand pear ‘Meirensu’ (*P*. *pyrifolia* Nakai) (MRS) were sampled from a commercial orchard in Kunming City, Yunnan Province, China. And the owner of the orchard gave permission to conduct our study on this site. We stated that no specific permissions were required for these locations/activities, because our field studies did not involve endangered or protected species. The MRS fruit were bagged at 40 days after full bloom (DAFB) with double layers of yellow-black paper bags. Half of the fruit were re-exposed to sunlight when the bags were removed 10 days before harvest, and the remaining fruit served as control samples. Thirty fruit were randomly sampled with three biological replicates at 0 H (hour), 6 H, 24 H, and 144 H after bag removal, while control fruit were sampled at the same times and were designated as 6 HC, 24 HC, and 144 HC (C, control; the 0 H sample did not require a control). Fruit peel was collected and immediately frozen in liquid nitrogen and stored at −80°C until use.

### Plant materials of bud dormancy

The bud materials used for the transcriptome analysis of ‘Suli’ pear (*P*. *pyrifolia* white pear group) and Japanese pear ‘Kosui’ (*P*. *pyrifolia*) have been described previously [13].

### Cytokinin treatment of fruitlets

Six mature ‘Cuiguan’ pear (*P*. *pyrifolia* Nakai) trees with similar tree vigor and loading were selected from the orchard of Zhejiang University, Hangzhou, Zhejiang, China. Receptacles of ‘Cuiguan’ flowers were sprayed with 30 mg/L CPPU/0.02% Tween-20 as the cytokinin treatment and with distilled water/0.02% Tween-20 as the negative control during the full-blossom period. Receptacles were collected at 0 d before treatment, and fruitlets were collected at 3 d, 7 d, 11 d, 16 d, 21 d, and 31 d after treatment. The pear peel was carefully collected and immediately frozen in liquid nitrogen and stored at −80°C until use.

### Identification of B-type RR genes in pear

B-type RR genes *(B-PpRR)* were isolated from the published genome sequence of *P*. *bretschneideri* (http://gigadb.org/dataset/100083, Wu et al. 2013). The hidden Markov model (HMM) profiles of the response regulator receiver domain (PF00072) and Myb-like DNA-binding domain (PF00249) were used as queries to search the white pear database using HMMER 3.0 (http://hmmer.org/) with default parameters [14–15]. The sequences containing both two domains were selected and compared with the *B-ARR* genes in the Plant Transcription Factor Database (http://planttfdb.cbi.pku.edu.cn/index.php) [16].

### Mapping *B-PpRR* genes on pear chromosomes

We searched for the locations of *B-PpRR* genes in the sequences of pear chromosomes from GigaDB (http://gigadb.org/dataset/100083) [17]. The figure was drawn using JoinMap4.0 software.

### Sequence alignment and phylogenetic analyses

The *B-PpRR* family sequences for *Arabidopsis*, apple, and strawberry were retrieved from the Plant Transcription Factor Database. The *B-PpRR* protein sequences were aligned using MEGA5.0 (http://www.megasoftware.net/download_form) with default settings [18]. Phylogenetic trees were constructed using MEGA 5.0 software with the neighbor-joining (NJ) method and 1000 bootstrap replicates.

### Gene structure and conserved motif identification

The gene structure of each *B-PpRR* gene was drawn using the online software Gene Structure Display Server (http://gsds.cbi.pku.edu.cn/) [19]. Motifs in pear B-PpRR protein sequences were identified using the motif-based sequence analysis tool, MEME Suite Version 4.11.2 [20]. The optimum width of amino acid sequence was set from 6 to 30. The maximum number of motifs was set to 30.

### Analysis of gene transcript levels during pear fruit development, bud dormancy, and anthocyanin accumulation

Gene transcript profiles during pear fruit development (not published), bud dormancy [13, 21], and anthocyanin accumulation (not published) were analyzed using the Tophat-Cufflinks Pipeline [22]. The figure was drawn using Heatmap Illustrator software, version 1.0 (http://hemi.biocuckoo.org/) [23].

### Measurements of anthocyanin

For anthocyanin extraction, fruit peel (1g) was placed in 10ml methanol: acetic acid (99:1, v/v). Extractions were performed overnight at 4°C. The absorbance at 530, 620 and 650 nm were measured using a DU800 spectrophotometer (Beckman Coulter, Fullerton, CA, USA). The anthocyanin content was normalized using the following formula: Normalized OD530 = [(OD_530_−OD_650_)−0.2 * (OD_650_−OD_620_)] [24].

### RNA extraction and cDNA synthesis

Total RNA was extracted using a modified CTAB method [25]. After digestion of genomic DNA by DNase I, the concentration of total RNA was measured using BioDrop (Biochrom). First-strand cDNA was synthesized from 4 μg DNA-free RNA using the Revert Aid^™^ First-Strand cDNA Synthesis Kit (Fermentas, Glen Burnie, MD, USA) according to the manufacturer’s instructions. The cDNA was diluted 10-fold and 2 μl was used as the template for gene cloning and real-time quantitative PCR (Q-PCR) analysis.

### Real-time quantitative PCR (Q-PCR)

Primers for real-time PCR analysis were designed with a primer design tool (http://www.ncbi.nlm.nih.gov/tools/primer-blast/index.cgi?LINK_LOC=BlastHome).

The efficiency and specificity of primers were determined by melting curves and by sequencing PCR products. Real-time PCR was performed using a CFX96 instrument (Bio-Rad). Each Q-PCR reaction mixture contained 7.5 μl SYBR Premix Ex TaqTM (Takara, Ohtsu, Japan), 0.5 μl each primer (10 μM), 1 μl cDNA, and 5.5 μl RNase-free water in a total volume of 15 μl. The reaction started with a preliminary step of 95°C for 3 min, followed by 40 cycles of 95°C for 10 s and 60°C for 30s. A template-free control for each primer pair was included for each run. For each gene, the relative transcript level was determined with the 2^-ΔΔCt^ algorithm [26] with normalization against the pear *Actin* gene.

### Statistical analysis

The LSD values (α = 0.05) were calculated for mean separations using Statistical Product and Service Solutions software v. 19 (SPSS Inc., Chicago, IL, USA).

## Results

### Identification, phylogenetic analysis, and structural analysis of B-PpRR proteins

In total, 13 genes putatively encoding *B-PpRR* proteins were isolated from the published genome sequence of *P*. *bretschneideri*. After manually removing duplicated sequences, the remaining 11 genes were further analyzed.

**Fig 1:**
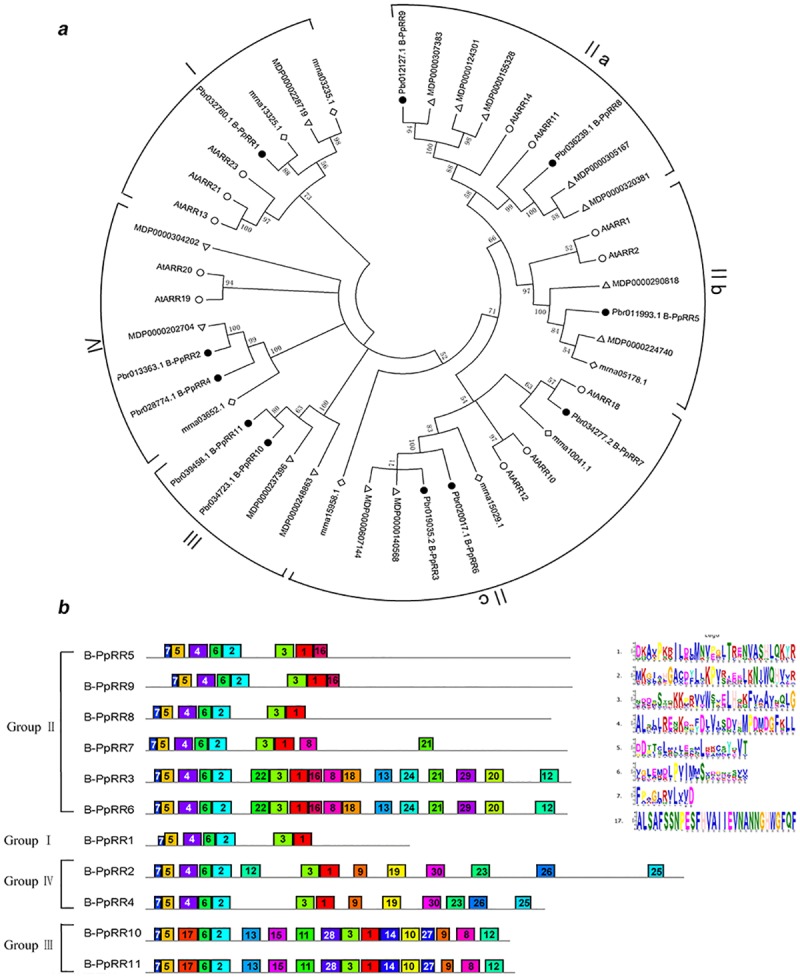
Phylogenetic and structural analysis of *B-PpRR* genes. a. Phylogenetic tree constructed with B-RR protein sequences from pear (B-PpRR), apple (MDP), strawberry (mma), and *Arabidopsis* (ARR). Protein sequences were obtained from the Plant Transcription Factor Database. Phylogenetic tree was constructed using MEGA5.0 by the neighbor-joining method with 1000 bootstrap replicates. B-PpRR proteins clustered into four groups. b. Distribution of conserved motifs in B-PpRR protein sequences. Motifs (1–30) were identified using MEME search tool. Most important motifs are listed. Length and order of each motif corresponds to actual length and position in protein sequence. Motifs 7, 5, 4/17, 6, and 2 correspond to REC signal receiver domain; motifs 1 and 3 correspond to MYB domain.

To evaluate the phylogenetic relationships of pear *B-PpRR* genes and classify them within established subfamilies, we compared the B-PpRR sequences from pear, apple (*Malus domestica*), strawberry (*Fragaria × ananassa*), and Arabidopsis (*Arabidopsis thaliana*). The phylogenetic analysis clustered the B-PpRR family into four groups, among which group II was further divided into three subgroups (Fig 1a).

The MEME motif analysis revealed that some of the motifs were conserved, while others were distributed among some of the subfamilies (Fig 1b). All the *B-PpRR* proteins had the REC signal receiver domain (motifs 7, 5, 4/17, 6, 2) at the N-terminal for signal reception in the two-component system. Groups I and III contained an OmpR domain consisting of REC and winged-helix (wHTH) domains, while the type of REC domain in group III differed from that in other groups. All of the *B-PpRR* genes contained the MYB domain (including motifs 1 and 3) so that all the *B-PpRR* genes were confirmed to be MYB transcription factors. However, the type of MYB domain in Group IV differed from that in other groups. Specifically, Group II contained a REC domain like that in Group IV and an MYB_SHAQKYF domain (Fig 1b). Some other motifs were found only in some members. For example, motif 8 was present in only five genes and motif 13 in only four genes. Notably, ARR1/10/12, which play essential (or general) roles in cytokinin signal transduction, were included in Group II.

### Chromosomal locations and structure of *B-PpRR* genes

**Fig 2:**
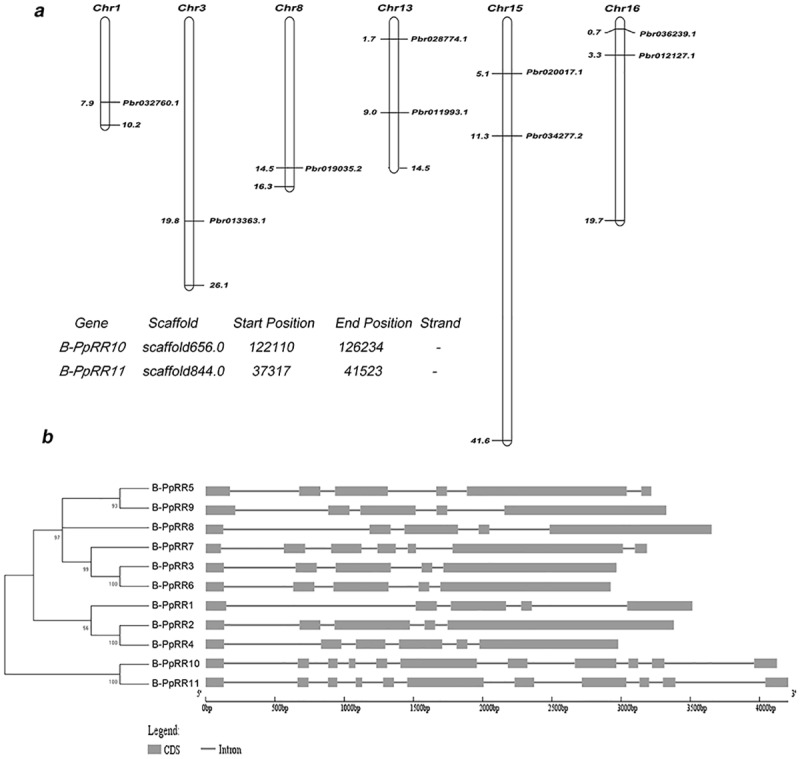
Chromosomal distribution and structures of *B-PpRR* genes in pear. a. Distribution of nine *B-PpRR* genes on six pear chromosomes; remaining two *B-PpRR* genes were located on scaffolds that have not been mapped to chromosomes. b. Gene structure of *B-PpRR* genes. Gray rectangles represent exons, lines represent introns.

We identified the locations of nine *B-PpRR* genes on pear chromosomes (Fig 2a). The remaining two genes were located on scaffolds that have not been mapped to chromosomes. The nine *B-PpRR* genes were unevenly distributed on six of the 17 pear chromosomes. A generic name from *B-PpRR1* to *B-PpRR11* was used to distinguish each of the *B-PpRR* genes in this study. The nomenclature of the *B-PpRR* genes was based on their position on pear chromosomes from 1 to 17, from top to bottom. The *B-PpRR* genes on scaffolds were named *B-PpRR10* and *B-PpRR11* based on their position on the scaffolds.

As gene structure is a typical imprint of evolution within a gene family, the structure of *B-PpRR* genes was displayed using tools at the GSDS website. Full-length cDNA sequences were compared with genomic DNA sequences to determine the numbers and positions of exons and introns in each *B-PpRR* gene. All of the *B-PpRR* genes in pear contained introns, the number ranging from four to 11 (Fig 2b). The number of introns was conserved within each group.

### Transcript profiles of *B-PpRR* genes during fruit development

**Fig 3:**
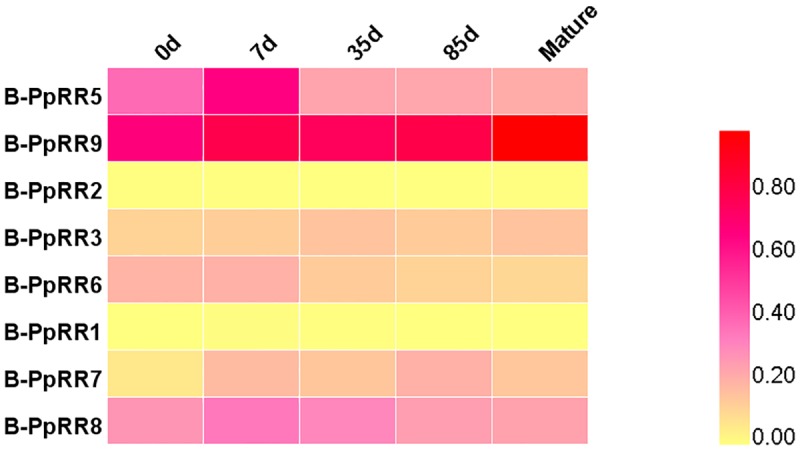
Transcript profile of *B-PpRR* genes in different development stages of different pears. Transcription profiles in different fruits were determined from transcriptome data. Transcript levels were normalized with min-max method. Samples were collected at 0, 7, 35, and 85 days after full bloom and maturity. XQ, *Pyrifolia* cv. Xueqing.

To investigate the potential functions of *B-PpRR* genes during fruit development, the transcript profiles of *B-PpRR* genes in large fruit of pear ‘Xueqing’ (XQ, average weight >400 g per fruit) (*P*. *pyrifolia*) in different developmental stages were determined from transcriptome data (Fig 3). Six *B-PpRR* genes, *B-PpRR5*/*9*/3/*6*/*7*/*8*, showed differences in their transcript levels among different developmental stages. Among them, *B-PpRR5*/*6/8* were transcribed in the early stages of fruit development and their transcript levels peaked at 7d after full blossom, probably because of the requirement to trigger and maintain cell division at anthesis until the ovary reached full maturity. There were high transcript levels at *B-PpRR9* at all time points and peak transcript levels at maturity, while the transcript level of *B-PpRR7* peaked at 85 d. Notably, all these *B-PpRR* genes belonged to group II, suggesting that these *B-PpRR* genes primarily participate in regulating fruit development.

### Transcript profiles of *B-PpRR* genes during floral bud dormancy

**Fig 4:**
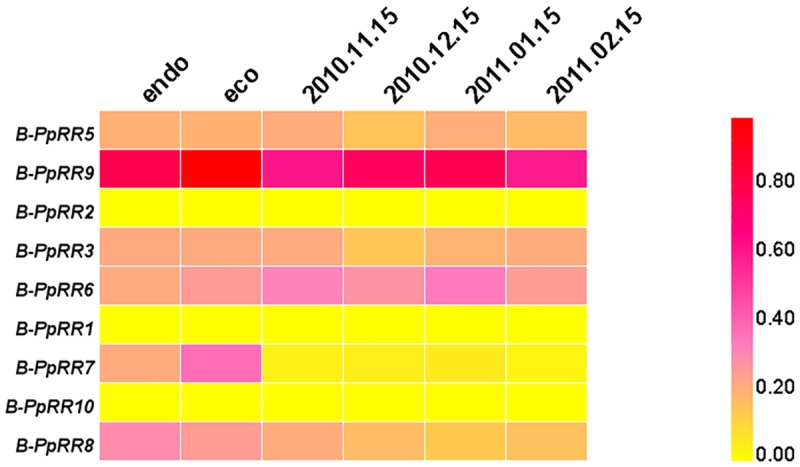
Transcript profiles of *B-PpRR* genes during bud dormancy. Transcription profiles during bud dormancy were determined from transcriptome data. Transcript levels were normalized with min-max method. Samples of *P*. *pyrifolia* white pear group cv. Suli were collected from Nov. 15 2011 to Feb. 15 2012. Transcription profiles of *P*. *pyrifolia* ‘Kosui’ corresponded to endo (endodormancy) and eco (transitional state of endodormancy and ecodormancy) stages.

The transcript profiles of *B-PpRR* genes were investigated in the floral buds of ‘Suli’ pear (*P*. *pyrifolia* white pear group) during dormancy [13] (Fig 4). *B-PpRR5*/*9*/3/6/8 were differentially transcribed among different dormancy stages. Among these five genes, *B-PpRR9* showed the highest transcript levels, which gradually increased during chilling accumulation and peaked towards endodormancy release. The transcript levels of *B-PpRR5* and *B-PpRR6* were low during chilling accumulation but peaked during endodormancy release, while the transcript levels of *B-PpRR8* showed the opposite trend. The transcript level of *B-PpRR3* decreased to minimum levels during chilling accumulation and then increased.

The transcript profiles of *B-PpRR* genes were also investigated in the dormant floral buds of Japanese pear ‘Kosui’ (*P*. *pyrifolia*) [21]. Four *B-PpRR* genes, *B-PpRR9*/*6*/*7*/*8*, showed significant differences in transcript levels among different stages. Among these genes, *B-PpRR9*/*6*/*7* showed increased transcript levels during endodormancy release, while *B-PpRR8* showed the opposite transcript profile. The transcript profiles of these genes in ‘Kosui’ pear were similar to those in ‘Suli’, suggesting their conserved functions in regulating bud dormancy in Chinese pear and Japanese pear.

### Transcript profiles of *B-PpRR* genes during anthocyanin accumulation

**Fig 5:**
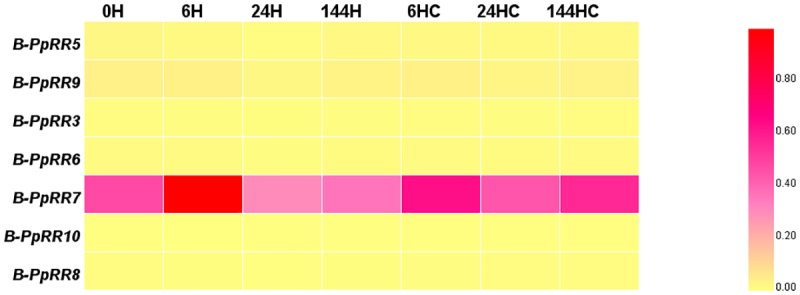
Heat maps showing transcriptional profiles of *B-PpRR* genes after removing bags from fruit of *P*. *pyrifolia* Nakai ‘Meirensu’. Gene transcript profiles were determined from transcriptome data. Transcript levels were normalized with min-max method.

Cytokinins have been reported to enhance anthocyanin content and transcript levels of anthocyanin biosynthetic genes in the presence of light in various plants. To verify the function of *B-PpRR* genes in regulating anthocyanin accumulation in pear, the transcript profiles of *B-PpRR* genes were investigated in the peel of bagged fruit of MRS using transcriptome data (under submission) (Fig 5). After removing the bags from fruit, anthocyanins accumulated in pear skin, but no anthocyanins accumulated in control fruit skin. Among the *B-PpRR* genes, *B-PpRR7* showed high transcript levels in all samples, particularly in the 6 H sample after bag removal, suggesting that *B-PpRR7* might participate in regulating light-induced anthocyanin accumulation. In contrast, the transcript levels of other *B-PpRR* genes were very low or even undetected.

**Fig 6:**
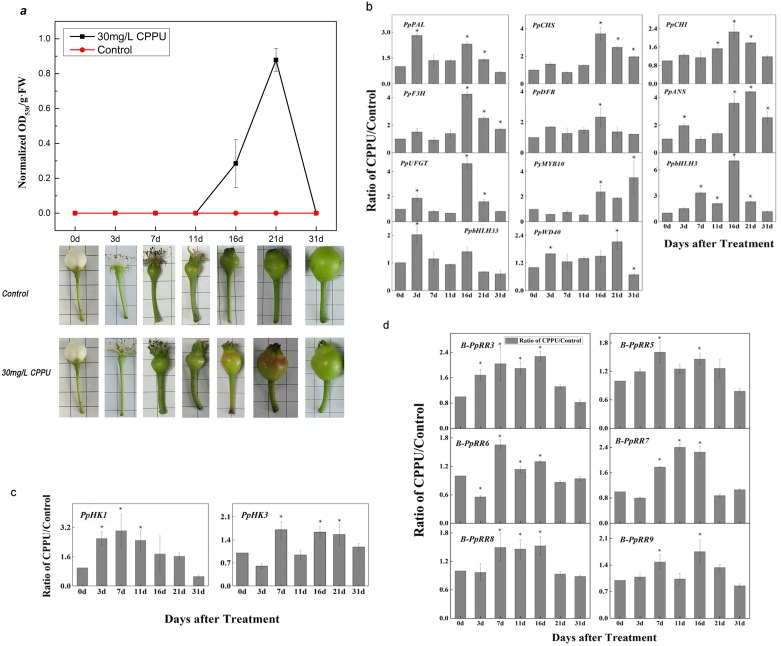
Anthocyanin induction in *P*. *pyrifolia cv*. Cuiguan by N-(2-chloro-4-pyridyl)- N′-phenylurea (CPPU) treatment. a. Anthocyanin accumulation and phenotypes of ‘Cuiguan’ at 0, 3, 7, 11, 16, 21, and 31 days after 30 mg/L CPPU or water treatment. b. Transcript levels of anthocyanin biosynthetic genes and regulatory genes. c. Transcript levels of cytokinin receptor genes. d. Transcript levels of group II *B-PpRR* genes after CPPU treatment. Values shown are mean ± standard error of three replicates.

To further verify the functions of cytokinin and *B-PpRR*s in regulating fruit coloration, the green/yellow pear cultivar ‘Cuiguan’ was treated with the cytokinin CPPU. Unlike most non-red apples whose fruit accumulate anthocyanin at the early developmental stage, most Asian pears never accumulate anthocyanin. After the CPPU treatment of receptacles during the full-blossom period, the fruitlets accumulated anthocyanin after 16 days, and the anthocyanin content peaked at 21 d after treatment (Fig 6a) while the control fruit remained green. The relative transcript levels of the anthocyanin biosynthesis genes *PpCHS*, *PpCHI*, *PpF3H*, *PpDFR*, and *PpUFGT*, peaked at 16 d after treatment, while that of the upstream gene *PpPAL* peaked at 3 d after treatment. The transcript level of *PpANS* peaked at 21 d after treatment. Among the regulator genes, *PyMYB10* was up-regulated from 16 d to 31 d after CPPU treatment and *PpbHLH3* transcript levels peaked at 16 d after CPPU treatment (at the same time as *PpWD40*), while the highest mRNA level of *PpbHLH33* was at 3 d after treatment. In general, the peak transcript levels of anthocyanin biosynthesis genes preceded the peak of visible color accumulation, indicating that CPPU triggered anthocyanin accumulation by activating anthocyanin biosynthetic genes.

We also analyzed the transcript levels of genes within the cytokinin signaling pathway. The transcript levels of *PpHK1* (cytokinin receptor gene) increased at 7 d after treatment with CPPU and then gradually declined, while the transcript levels of *PpHK3* peaked at 7 d and fluctuated until 16 d after treatment (Fig 6c). These results indicated that CPPU activation of the cytokinin signaling pathway mainly depended on *PpHK1*.

Finally, the transcript levels of *B-PpRR* genes in group II, *B-PpRR5/9/3/6/7/8* were analyzed by q-PCR assays. For most of these genes, their transcript levels first increased and then decreased (Fig 6d). Specifically, CPPU induced transcription of *B-PpRR3* and *B-PpRR7*. Their transcript profiles were similar to that of *PpHK1*, suggesting that they played important roles in regulating anthocyanin biosynthesis under these conditions.

## Discussion

*B-RR* genes have been characterized in *Arabidopsis* [4], rice [27], and poplar [28]. In this study, 11 *B-RR* genes were identified from the genome sequence of *P*. *bretschneideri* (Fig 1a), approximately equivalent to those in *Arabidopsis* (12 genes) [4], rice (13 genes) [27] and poplar (11 genes) [28], suggesting that no *B-RR* gene family expansion in these species although some genome duplication events happened in some species, such as pear (Wu et al, 2013). The importance of cytokinins in plant growth and development has probably led to the conserved family size of *B-RR* genes. This may reflect selection against substantial changes in the stoichiometry of *B-RR* genes in the cytokinin signaling pathway [28].

The REC signal receiver domain at the N-terminal and the MYB domain were present in all of the pear B-type RR genes (Fig 1b), indicating that these genes are MYB transcription factors and that their N-terminal is conserved. The *B-PpRR* genes were further classified into four subgroups according to the phylogenetic and structural analyses (Fig 1a). In *Arabidopsis*, B-ARRs formed three groups based on a phylogenetic analysis [29]. The extra group in pear was group III, whose members had a different REC domain from that in other groups, which haven’t been distinguished in *Arabidopsis* (Fig 1b).

Interestingly, members of Group II showed differences in their transcript levels during fruit development (Fig 3), bud dormancy (Fig 4), and anthocyanin accumulation induced by light (Fig 5) or cytokinins(Fig 6d), suggesting their involvement in these physiological processes. In *Arabidopsis*, the well-studied *ARR1/10/12*, were also clustered in Group II. The *arr1 arr10 arr12* triple mutant showed almost complete insensitivity to exogenously applied cytokinins, which exhibited decreased cell division in the shoot, enhanced seed size, increased sensitivity to light, altered chlorophyll and anthocyanin concentrations, and an aborted primary root with with protoxylem but no metaxylem [6]. Our results also showed that that Group II members played dominant roles in cytokinin signal transduction in pear. The functions of the other three groups are yet to be determined.

### *B-PpRR* links cytokinin signaling pathway and fruit development

In most plants, fruit development can be divided into four phases. The first phase is ovary development and fruit set; the second phase is fruit growth via cell division, which determines the number of cells; the third phase begins after cell division ceases, and consists of cell expansion until the fruit reaches its final size; and the last phase is fruit ripening [30]. We analyzed the transcript profiles of *B-PpRR* genes in large XQ pear fruit (>400 g) during its development that contains the four phases (Fig 3). We found that five *B-PpRR* genes showed changes in their transcript levels during fruit development, among which *B-PpRR*5/6/8 showed high transcript levels at the early stage, when there was rapid cell division (Fig 3). As cytokinins induced the cell division in the early development of pear fruit, the spatial and temporal synthesis and action of cytokinins were required for the early development of fruit growth [31], the high expression of *B-PpRR* genes may contribute the active cytokinin responses in these stages. Indeed, the involvement of *B-RR* genes in the regulation of cell division [32], which is directly related to the cell number of fruits, has been well characterized. Specifically, in *Arabidopsis*, *ARR1* and *ARR12* activate *SHY2*, a repressor of auxin signaling, which then affects the cell differentiation and division of the root meristem [8–9]. In addition, the triple mutant of *arr1/10/12* also had far fewer cells in the root, compared with that in wild type [7]. These findings suggest that *B-RR* genes participate in regulating cell number and organ size. In our study, it is likely that *B-PpRR*5/6/8 were the main *B-PpRR* genes mediating cytokinin signals in the early stage of pear fruit growth (Fig 3), which indicated that *B-PpRR*5/6/8 may play important roles in the fruit size of XQ pear. In contrast, *B-PpRR*9 showed higher transcript levels at the later stage of pear fruit development, and its transcript levels were higher than those of all other *B-PpRR* genes throughout fruit development (Fig 3). This transcript profile suggested that *B-PpRR*9 may have much more diverse functions during fruit development and may also be involved in seed development during fruit ripening, as reported in tea and soybean [33–34]

### Cytokinin regulates bud dormancy through *B-PpRR* genes

Dormancy is a beneficial biological property which is essential for survival in a hostile environment. It is a complex process that is regulated by photoperiod, temperature, and phytohormones. In pear, plant hormones are important factors in regulating dormancy [35]. According to the transcription profiles, we found that several *B-PpRR* genes showed changes in their transcript levels during dormancy in both Chinese white pear and Japanese pear (Fig 4). Specifically, *B-PpRR9*/*6* in both cultivars, *B-PpRR5*/3 in ‘Suli’, and *B-PpRR7* in ‘Kosui’, showed similar trends in their transcription profiles during the transitional state of endodormancy and ecodormancy, and their transcript levels increased after endodormancy release (Fig 4). These results suggested that *B-RR*-mediated pathway probably contributed to the release of endodormancy. Although the effects of cytokinin on the regulation of endodormancy of deciduous trees have not been well described, the positive function of cytokinin on the termination of potato tuber dormancy has been reported [36]. Furthermore, Rousselin et al. (1992) also found that a cytokinin-resistant mutant of *N*. *plumbaginifolia* exhibited reduced seed dormancy [37], indicating that cytokinins play vital roles in dormancy. Similarly, our findings suggested that the cytokinin-signaling pathway is involved in regulating pear bud dormancy. However, *B-PpRR8* showed the opposite trend in its transcript profile during the transitional state of endodormancy and ecodormancy, and its transcript levels decreased after endodormancy release (Fig 4). Together, these results illustrated the spatio-temporal and tissue-specific transcription patterns of *B-PpRRs* in pear bud dormancy.

### Cytokinins enhance anthocyanin biosynthesis via *B-PpRR* genes in pear

Anthocyanin accumulation is has been known as affected by various hormones, including cytokinin [38]. Exogenous cytokinin has also been shown to induce anthocyanin accumulation in strawberry suspension cells [39], *A*. *thaliana* [40], and in callus cultures of red-fleshed apple [41]. In this work, we confirmed that anthocyanin biosynthesis was enhanced by application of cytokinin in the pear fruitlet (Fig 6a) in which *B-PpRR* genes were involved. Among the *B-PpRR*s, *B-PpRR7* showed peak transcript levels immediately after bag removal (Fig 5), indicating that it is an early light-responsive gene that is possibly involved in light-induced anthocyanin accumulation. However, it seemed that *B-PpRR7* was not able to directly regulate anthocyanin biosynthesis because the peak transcript level of *B-PpRR7* occurred much earlier than that of anthocyanin accumulation (Fig 5). Interestingly, the transcriptional pattern of *B-PpRR7* was similar to that of *PpHY5*, the key transcriptional factor in light signal transduction, suggesting a potential interaction between these two genes. In *Arabidopsis*, cytokinin induced anthocyanin accumulation in a light-dependent manner and resulted in the increased accumulation of HY5 [42]. It is possible that *B-PpRR7* is involved in light-induced anthocyanin biosynthesis via directly or indirectly inducing *PpHY5*.

Furthermore, the application of CPPU on ‘Cuiguan fruitlet’ induced the expression of most anthocyanin biosynthetic genes as well as the regulator gene *PyMYB10* (Fig 6b), indicating that cytokinin induced anthocyanin accumulation via *PyMYB10*. Similarly, in *Arabidopsis*, exogenous cytokinin also enhanced anthocyanin accumulation via up-regulation of *PAP1* and *bHLHs* [10] through the up-regulation of a subset of *B-ARR* genes, which was also observed in our results (Fig 6d). Taken together, cytokinin enhance the anthocyanin accumulation through the activation of anthocyanin-biosynthesis genes via *B-PpRR* genes in pear.

Interestingly, the CPPU-induced anthocyanin accumulation was only observed in fruitlets (Fig 6a) but not in mature fruit, probably because anthocyanin biosynthesis in fruitlets and ripe fruit is controlled by different regulatory mechanisms. In grape, two kinds of MYB transcription factors that regulated phenylpropanoid biosynthesis showed different expression patterns in fruitlets and ripe fruit; for example, *VLMybA1-1* was detected in ripening grape, and *VvMYB5a* was detected in the fruitlets [43–44]. Similarly, therefore, the *B-PpRR* family of MYB transcription factors might directly regulate the biosynthesis of anthocyanin in ‘Cuiguan’ pear fruitlets. It is also an interesting finding that CPPU induced coloration in a non-red pear cultivar. This may be a useful strategy to alter fruit color.

## Conclusion

The results of this study showed that cytokinins are involved in regulating pear fruit size and development, and pear bud dormancy, via *B-PpRR* genes. The results also showed that cytokinins play positive roles in light- or hormone-induced anthocyanin accumulation. Notably, all the differentially transcribed *B-PpRR* genes belong to group II, indicating that this group of *B-PpRR* genes is involved in the cytokinin signaling pathway. Our results also indicated that the functions of *B-RR* genes are conserved among different species. Further research is required to explore the molecular mechanism of cytokinin-induced anthocyanin accumulation.

## Supporting information

S1 File Primers for real-time PCR analysis in this work. (XLSX)

S2 File List of accession numbers of RNA-seq data used in this work. (XLSX)

## References

[1] Muller B, Sheen J. Advances in cytokinin signaling. Science. 2007; 318(5847):68–69. 10.1126/science.1145461 17916725

[2] Kakimoto T. Perception and signal transduction of cytokinins. Annu Rev Plant Biol. 2003; 54(1):605–627. 14503005 10.1146/annurev.arplant.54.031902.134802

[3] Mizuno T. Compilation of all genes encoding two-component phosphotransfer signal transducers in the genome of *Escherichia coli*. DNA Res. 1997; 4(2):161–8. 9205844 10.1093/dnares/4.2.161

[4] D'Agostino IB, Deruère J, Kieber JJ. Characterization of the response of the *Arabidopsis* response regulator gene family to cytokinin. Plant Physiol. 2000; 124(4):1706–1717. 11115887 10.1104/pp.124.4.1706 PMC59868

[5] Hwang I, Sheen J. Two-component circuitry in *Arabidopsis* cytokinin signal transduction. Nature. 2001; 413(6854):383–389. 10.1038/35096500 11574878

[6] Argyros RD, Mathews DE, Chiang YH, Palmer CM, Thibault DM, Etheridge N, et al Type B response regulators of *Arabidopsis* play key roles in cytokinin signaling and plant development. Plant Cell. 2008; 20(8):2102–16. 10.1105/tpc.108.059584 18723577 PMC2553617

[7] Ishida K, Yamashino T, Yokoyama A, Mizuno T. Three type-B response regulators, ARR1, ARR10 and ARR12, play essential but redundant roles in cytokinin signal transduction throughout the life cycle of *Arabidopsis thaliana*. Plant Cell Physiol. 2008; 49(1):47–57. 10.1093/pcp/pcm165 18037673

[8] Ioio RD, Nakamura K, Moubayidin L, Perilli S, Taniguchi M, Morita M, et al A genetic framework for the control of cell division and differentiation in the root meristem. Science. 2008; 322(5906):1380–1384. 10.1126/science.1164147 19039136

[9] Moubayidin L, Perilli S, Dello Ioio R, Di Mambro R, Costantino P, Sabatini S. The rate of cell differentiation controls the *Arabidopsis* root meristem growth phase. Curr Biol. 2010; 20(12):1138–1143. 10.1016/j.cub.2010.05.035 20605455

[10] Das PK, Shin DH, Choi SB, Yoo SD, Choi G, Park YI. Cytokinins enhance sugar-induced anthocyanin biosynthesis in *Arabidopsis*. Mol Cells. 2012; 34(1):93–101. 10.1007/s10059-012-0114-2 22699753 PMC3887782

[11] Riou-Khamlichi C, Huntley R, Jacqmard A, Murray JA. Cytokinin activation of *Arabidopsis* cell division through a D-type cyclin. Science. 1999; 283(5407):1541–4. 10066178 10.1126/science.283.5407.1541

[12] Zhang C, Tanabe K, Wang S, Tamura F, Yoshida A, Matsumoto K. The impact of cell division and cell enlargement on the evolution of fruit size in *Pyrus pyrifolia*. Ann Bot-London. 2006; 98(3):537–543. 10.1093/aob/mcl144 PMC2803567 16845135

[13] Liu G, Li W, Zheng P, Xu T, Chen L, Liu D, et al Transcriptomic analysis of 'Suli' pear (*Pyrus pyrifolia* white pear group) buds during the dormancy by RNA-Seq. BMC GENOMICS. 2012; 13(700):700. 23234335 10.1186/1471-2164-13-700 PMC3562153

[14] Finn RD, Coggill P, Eberhardt RY, Eddy SR, Mistry J, Mitchell AL, et al The Pfam protein families database: towards a more sustainable future. Nucleic Acids Res. 2016; 44(D1):D279–D285. 10.1093/nar/gkv1344 26673716 PMC4702930

[15] Mistry J, Finn RD, Eddy SR, Bateman A, Punta M. Challenges in homology search: HMMER3 and convergent evolution of coiled-coil regions. Nucleic Acids Res. 2013; 41(12):e121–e121. 10.1093/nar/gkt263 23598997 PMC3695513

[16] Jin J, Zhang H, Kong L, Gao G, Luo J. PlantTFDB 3.0: a portal for the functional and evolutionary study of plant transcription factors. Nucleic Acids Res. 2013; 42(D1):D1182–D1187. 24174544 10.1093/nar/gkt1016 PMC3965000

[17] Wu J, Wang Z, Shi Z, Zhang S, Ming R, Zhu S, et al The genome of the pear (*Pyrus bretschneideri* Rehd.). Genome Res. 2013; 23(2):396–408. 10.1101/gr.144311.112 23149293 PMC3561880

[18] Tamura K, Peterson D, Peterson N, Stecher G, Nei M, Kumar S. MEGA5: molecular evolutionary genetics analysis using maximum likelihood, evolutionary distance, and maximum parsimony methods. Mol Biol Evol. 2011; 28(10):2731–9. 10.1093/molbev/msr121 21546353 PMC3203626

[19] Hu B, Jin J, Guo AY, Zhang H, Luo J, Gao G. GSDS 2.0: an upgraded gene feature visualization server. Bioinformatics. 2015; 31(8):1296–1297. 10.1093/bioinformatics/btu817 25504850 PMC4393523

[20] Bailey TL, Gribskov M. Combining evidence using p-values: application to sequence homology searches. Bioinformatics. 1998; 14(1):48–54. 9520501 10.1093/bioinformatics/14.1.48

[21] Bai S, Saito T, Sakamoto D, Ito A, Fujii H, Moriguchi T. Transcriptome analysis of Japanese pear (*Pyrus pyrifolia* Nakai) flower buds transitioning through endodormancy. Plant Cell Physiol. 2013; 54(7):1132–1151. 10.1093/pcp/pct067 23624675

[22] Trapnell C, Roberts A, Goff L, Pertea G, Kim D, Kelley DR, et al Differential gene and transcript expression analysis of RNA-seq experiments with TopHat and Cufflinks. Nat Protoc. 2012; 7(3):562–78. 10.1038/nprot.2012.016 22383036 PMC3334321

[23] Deng W, Wang Y, Liu Z, Cheng H, Xue Y. HemI: a toolkit for illustrating heatmaps. PLoS One. 2014; 9(11):e111988 10.1371/journal.pone.0111988 25372567 PMC4221433

[24] Bai S, Saito T, Honda C, Hatsuyama Y, Ito A, Moriguchi T. An apple B-box protein, MdCOL11, is involved in UV-B- and temperature-induced anthocyanin biosynthesis. Planta. 2014; 240(5):1051–1062. 10.1007/s00425-014-2129-8 25074586

[25] Zhang D, Yu B, Bai JH, Qian MJ, Shu Q, Su J, et al Effects of high temperatures on UV-B/visible irradiation induced postharvest anthocyanin accumulation in 'Yunhongli No. 1' (Pyrus pyrifolia Nakai) pears. Sci Hortic. 2012; 134(53–59.

[26] Livak KJ, Schmittgen TD. Analysis of relative gene expression data using real-time quantitative PCR and the 2−ΔΔ^CT^ method. Methods. 2001; 25(4):402–8. 10.1006/meth.2001.1262 11846609

[27] Schaller GE, Doi K, Hwang I, Kieber JJ, Khurana JP, Kurata N, et al Nomenclature for two-component signaling elements of rice. Plant Physiol. 2007; 143(2):555–7. 10.1104/pp.106.093666 17284581 PMC1803756

[28] Ramirez-Carvajal GA, Morse AM, Davis JM. Transcript profiles of the cytokinin response regulator gene family in *Populus* imply diverse roles in plant development. New Phytol. 2008; 177(1):77–89. 10.1111/j.1469-8137.2007.02240.x 17944821

[29] Mason MG. Type-B response regulators display overlapping expression patterns in *Arabidopsis*. Plant Physiol. 2004; 135(2):927–937. 10.1104/pp.103.038109 15173562 PMC514127

[30] Gillaspy G, Ben-David H, Gruissem W. Fruits: a developmental perspective. Plant cell. 1993; 5(10):1439–1451. 10.1105/tpc.5.10.1439 12271039 PMC160374

[31] Matsuo S, Kikuchi K, Fukuda M, Honda I, Imanishi S. Roles and regulation of cytokinins in tomato fruit development. J Exp Bot. 2012; 63(15):5569–79. 10.1093/jxb/ers207 22865911 PMC3444270

[32] Mok ADW, Mok MC. Cytokinin metabolism and action. Annu Rev Plant Biol. 2001; 52(2):89–118. 10.1146/annurev.arplant.52.1.89 11337393

[33] Crosby KE, Aung LH, Buss GR. Influence of 6-Benzylaminopurine on fruit-set and seed development in two soybean, *Glycine max* (L.) Merr. genotypes. Plant Physiol. 1981; 68(5):985–8. 16662076 10.1104/pp.68.5.985 PMC426030

[34] Bhattacharya A, Nagar PK. Changes in endogenous cytokinin activity during seed development in tea. Indian J Plant Physi. 2006; 11(3):287–290.

[35] Koornneefa M, Bentsinka L, Hilhorstb H. Seed dormancy and germination. Curr Opin Plant Biol. 2002; 5(1):33–36. 11788305 10.1016/s1369-5266(01)00219-9

[36] Suttle JC. Postharvest changes in endogenous cytokinins and cytokinin efficacy in potato tubers in relation to bud endodormancy. Physiol Plantarum. 1998; 103(1):59–69.

[37] Rousselin P, Kraepiel Y, Maldiney R, Miginiac E, Caboche M. Characterization of three hormone mutants of *Nicotiana plumbaginifolia*: evidence for a common ABA deficiency. Theor Appl Genet. 1992; 85(2–3):213–21. 10.1007/BF00222862 24197307

[38] Jaakola L. New insights into the regulation of anthocyanin biosynthesis in fruits. Trends Plant Sci. 2013; 18(9):477–83. 10.1016/j.tplants.2013.06.003 23870661

[39] Mori T, Sakurai M, Seki M, Furusaki S. Use of auxin and cytokinin to regulate anthocyanin production and composition in suspension cultures of strawberry cell. J Sci Food Agr. 1994; 65(3):271–276.

[40] Deikman J, Hammer PE. Induction of anthocyanin accumulation by cytokinins in *Arabidopsis thaliana*. Plant Physiol. 1995; 108(1):47–57. 12228453 10.1104/pp.108.1.47 PMC157304

[41] Ji X, Wang Y, Zhang R, Wu S, An M, Li M, et al Effect of auxin, cytokinin and nitrogen on anthocyanin biosynthesis in callus cultures of red-fleshed apple (*Malus sieversii* f.*niedzwetzkyana*). Plant Cell Tiss Org. 2015; 120(1):325–337.

[42] Vandenbussche F, Habricot Y, Condiff AS, Maldiney R, Van der Straeten D, Ahmad M. HY5 is a point of convergence between cryptochrome and cytokinin signalling pathways in *Arabidopsis thaliana*. Plant J. 2007; 49(3):428–441. 10.1111/j.1365-313X.2006.02973.x 17217468

[43] Deluc L. Characterization of a grapevine R2R3-MYB transcription factor that regulates the phenylpropanoid pathway. Plant Physiol. 2006; 140(2):499–511. 10.1104/pp.105.067231 16384897 PMC1361319

[44] Kobayashi S, Ishimaru M, Hiraoka K, Honda C. *Myb*-related genes of the Kyoho grape (*Vitis labruscana*) regulate anthocyanin biosynthesis. Planta. 2002; 215(6):924–933. 10.1007/s00425-002-0830-5 12355152

